# Cardiac Abnormalities in Relation to the Disease Activity Index Among Systemic Lupus Erythematosus Patients in a Tertiary Hospital: A Cross-Sectional Study

**DOI:** 10.7759/cureus.49495

**Published:** 2023-11-27

**Authors:** Sheila Attuquayefio, Alfred Doku, Dzifa Dey, Franscis Agyekum, Florence K Akumiah, Anthony G Kweki, Uchenna M Amaechi, Henry O Aiwuyo

**Affiliations:** 1 Internal Medicine, University of Ghana Medical Centre, Accra, GHA; 2 Internal Medicine, Korle-Bu Teaching Hospital, Accra, GHA; 3 Internal Medicine and Therapeutics, University of Ghana Medical School, Accra, GHA; 4 Internal Medicine, University of Ghana Medical School, Accra, GHA; 5 Internal Medicine/Cardiology, Colchester Hospital, East Suffolk and North Essex NHS Foundation Trust (ESNEFT), Colchester, GBR; 6 Internal Medicine, Lagos University Teaching Hospital, Lagos, NGA; 7 Internal Medicine, Brookdale University Hospital and Medical Center, Brooklyn, USA

**Keywords:** disease activity index, immune complex deposition, autoantibody production, autoimmune disease, cross-sectional study, systemic lupus erythematosus (sle), echocardiographic

## Abstract

Background: Systemic lupus erythematosus (SLE) is a multisystem autoimmune connective tissue disorder involving multiple organs and systems. Cardiovascular involvement in SLE patients is a major cause of morbidity and mortality. Although subclinical cardiac abnormalities exist among SLE patients, they are rarely screened for. Echocardiography has been demonstrated to be a useful tool for the early diagnosis of cardiac abnormalities in SLE patients, many of which are clinically silent. Early recognition of cardiovascular abnormalities is vital for the prompt initiation of the appropriate management. This study aims to determine the prevalence of various structural and functional cardiac abnormalities among SLE patients and to determine its association with the modified SLE Disease Activity Index 2000 (modified SLEDAI-2K).

Methods: The study was a cross-sectional study of SLE patients at the Korle-Bu Teaching Hospital (KBTH), Accra, Ghana, from June to December 2021. The setting was the rheumatology outpatient clinic of the KBTH and included adult men and women, 18 years and above, diagnosed with SLE with no known cardiac abnormalities. The baseline demographic and clinical characteristics of the participants were determined. A detailed transthoracic echocardiogram was performed for all patients. The frequency of common cardiac pathologies was determined and compared between those with a high modified SLEDAI-2K and those with a low modified SLEDAI-2K.

Results: Ninety-nine SLE patients participated in the study with a mean age of 35.12 years. Females formed the majority (90.9%) of the participants. The mean age at diagnosis of SLE was 28.7 years and the mean disease duration was 4.6 years. All of the participants were on at least two disease-modifying medications. The mean modified SLEDAI-2K score was 9.1. Thirty-five percent (35%) of the patients had mild to moderately active disease and 39% had severely active disease. Sixty-six (66%) out of the severely active disease group had abnormal echocardiographic findings, while 28% of those with mild to moderate disease had abnormal echocardiographic findings. Echocardiographic abnormalities were found in 56 patients (47%), out of which 8.7% had valvular involvement, 15.7% had diastolic dysfunction, 5.2% had left ventricular hypertrophy (LVH), and 0.9% had left ventricular systolic dysfunction (LVSD). About 12% of the participants had pulmonary hypertension and 1% had pericardial involvement. The odds of echocardiographic abnormalities were 13.7 times higher in SLE patients with high disease activity compared to those with low disease activity (odds ratio (OR) = 13.714, 95% confidence interval (CI) = 3.804-49.442, p < 0.001). There was no significant association between cardiac abnormalities and SLE duration. No significant correlation between cardiac abnormalities and modified SLEDAI-2K score was found.

Conclusion: Cardiac abnormalities, especially left ventricular diastolic dysfunction (LVDD), valvular involvement, and pulmonary hypertension, are common in SLE patients. For SLE patients, especially those with active diseases, echocardiographic assessment should be considered in the management of SLE patients to enable early detection of cardiac abnormalities, early treatment, and thus a decrease in morbidity and mortality associated with cardiovascular involvement in SLE patients.

## Introduction

Systemic lupus erythematosus (SLE) is generally defined as a chronic multisystem, autoimmune disorder. It is identified by autoantibody production and immune complex deposition, which is directed against several organ systems, leading to significant morbidity and mortality [1]. SLE is a disease of unknown aetiology and diverse clinical presentation [2]. The course of lupus is typically unpredictable, characterized by flares and remissions. Over time, progressive damage severely negatively impacts the quality of life and organ function [3-5]. The clinical picture of this illness varies greatly from person to person and can affect numerous cells, tissues, and organs [6]. The joints, skin, mucous membranes, brain, kidneys, and heart are among the organ systems frequently affected in lupus patients [2]. The diagnosis of lupus is based on multisystem clinical and laboratory findings [7].

One of the main causes of morbidity and mortality in this patient population of SLE is cardiac involvement [8]. Studies have shown that variations in echocardiographic parameters that affect the structure and function of the heart, such as left atrial dilatation (LAD), left ventricular diastolic dysfunction (LVDD), and ventricular systolic dysfunction, are important features in SLE patients [9]. Cardiac involvement as a first symptom of SLE is uncommon; however, more than half of the time, cardiac involvement is linked to substantial morbidity and mortality [10].

Evidence of cardiac involvement is identified in up to 40% of autopsy studies, while only 10% of patients are clinically diagnosed [10]. Echocardiography is a helpful and non-invasive tool for evaluating alterations in heart structure and function, which can result in early intervention and lower cardiac morbidity and mortality in lupus patients. The majority of these cardiac manifestations are often mild and asymptomatic. Although several cutting-edge cardiac imaging modalities, including magnetic resonance imaging (MRI) and cardiac computed tomography (CT scan), are currently available and being used in the clinical management of patients with cardiac diseases, echocardiography remains the most used imaging technique, particularly in sub-Saharan Africa. This is because it is a cheaper, repeatable, non-invasive, and non-radioactive method.

As a clinical indicator for evaluating lupus disease activity over the previous 10 days, the Systemic Lupus Erythematosus Disease Activity Index (SLEDAI) was created and introduced globally in 1985 [11]. It is based on 24 questions that evaluate the clinical signs and symptoms of SLE, including physical signs and laboratory results from multiple organ systems [11]. The SLEDAI-2K is the updated 2002 version of the original SLEDAI. It has 24 self-explanatory components with fixed weights for various manifestations, with a maximum score of 105 and weights ranging from 1 to 8 [12]. The SLEDAI scores/activity categories are defined as follows [12]: no activity (SLEDAI = 0), mild activity (1-5), moderate activity (6-10), high activity (11-19), and very high activity (≥20).

In environments with limited resources, it is advantageous to assess disease activity using clinical rather than laboratory measures [13]. The SLEDAI-2K is reliable, accurate, sensitive, and responsive to change over time [11,14]. It is extremely practical and widely employed for clinical and research purposes [11,14]. The modified SLEDAI-2K that is used in this study is calculated by omitting the immunologic variables, therefore making it less expensive [12]. The revised Systemic Lupus Activity Measure (SLAM-R), Mexican Systemic Lupus Erythematosus Disease Activity Index (mMex-SLEDAI), and modified SLEDAI-2K are acceptable substitutes for the SLEDAI-2K for assessing disease activity in patients with lupus around the world, in either clinical or research settings, according to a study by Uribe et al. [12] The modified SLEDAI-2K demonstrated better metric properties at a cheaper cost and may therefore be the best alternative of all, particularly when dealing with disadvantaged populations.

One of the most difficult tasks for clinicians managing SLE patients is forecasting the disease progression and preventing permanent organ damage, both impacting patient outcomes. Cardiac diseases contribute significantly to morbidity and mortality among patients with SLE. However, many have subclinical cardiac abnormalities that are not screened for. Recently, there have been suggestions that echocardiography can be used as a screening tool to detect subclinical cardiac abnormalities for early risk stratification and optimization of clinical management [2]. However, this is not the standard of practice in sub-Saharan Africa. Recent studies have been done in sub-Saharan Africa to ascertain the epidemiology of SLE. However, few of these studies have looked at the frequency of cardiovascular abnormalities in this subregion, especially using a non-invasive method, such as 2D echocardiography, and its relationship with the modified SLEDAI-2K.

This study, therefore, seeks to determine the prevalence of echocardiographic abnormalities among patients with SLE and evaluate its relationship with the modified SLEDAI-2K.

## Materials and methods

The study is a cross-sectional study of patients diagnosed with SLE attending the Korle-Bu Teaching Hospital (KBTH), Accra, Ghana, from June to December 2021. The study was conducted at the rheumatology outpatient clinic of KBTH. The clinic is held once weekly at the Department of Medicine and sees an average of 41 SLE follow-up cases per week and about three new cases weekly. SLE patients form about 30% of all cases seen at the Rheumatology Clinic. KBTH is the main referral center for all hospitals within the Greater Accra region and for the entire Republic of Ghana.

Patients included in the study are adult patients, 18 years and above, with diagnoses of SLE (both follow-up and new cases), while adults with pre-existing cardiac diseases; pregnant women; patients with severe multiple organ dysfunction, such as respiratory and renal failure; and patients with cancer were excluded from the study.

The study began after the approval was given by the KBTH Ethics and Protocol Review Committee with approval number KBTH-IRB/000130/2020. The study participants were given complete information and reasons for the study. The information was presented in a language well understood by the participants. All benefits or risks associated with the study were clearly explained to the participants. The participants were also informed that their enrollment into the study was completely voluntary and that the standard of care provided at the clinic would not be jeopardized if they chose to participate in the study or not. Informed written consent was obtained in hard copy forms from participants who voluntarily agreed to enroll on the study. The forms were dated and signed before enrollment in the study.

Based on a study in Nigeria [15], the prevalence of SLE patients (attending a rheumatology clinic) is 5.28%. Therefore, using a prevalence of 5.28%, a Z score at 95% confidence level (1.96), and a level of significance of 0.05, the sample size was calculated to be 85.

Data were collected using a structured questionnaire that contained baseline demographic characteristics and risk factors for cardiovascular disease and the modified SLEDAI-2K.

All echocardiograms were performed by the principal investigator with the GE Vivid T8 ultrasound machine (GE Healthcare, USA), using the cardiac sector probe 3Sc-RS (1.3-4.0 MHz) with the capability of the M-mode, 2D, Doppler, transesophageal echocardiogram (TEE), and strain analysis.

Pericardial effusion was defined as echo-free space surrounding the heart and persistent throughout the cardiac cycle, while pericardial thickening is defined as thickness greater than 3 mm. The cutoff for systolic dysfunction was a fractional shortening of less than 29% and/or a left ventricular ejection fraction of less than 50%. Diastolic function was defined using mitral flow velocities, early mitral flow deceleration time (DT), and isovolumetric relaxation time (IVRT).

Diastolic dysfunction was graded as follows using the following parameters [16]: Grade 1 implies impaired relaxation (mitral E/A < 1, DT > 200 msec, IVRT > 100 msec), Grade 2 as pseudonormal pattern (E/A 0.8-1.5, DT 150-200 msec, IVRT < 60 msec and reversible on the Valsalva maneuver), and Grade 3 as restrictive reversible (E/A > 2, DT > 160 msec, IVRT < 60 msec and reversible on the Valsalva maneuver).

LV systolic dysfunction was defined as LV ejection fraction (LVEF) of <54% for women and <52% for men. LVH is defined as an increased left ventricular mass index (LVMI) greater than 95 g/m^2^ in women and an increased LVMI greater than 115 g/m^2^ in men.

Valvular thickening was defined as thickening greater than 3 mm for the mitral valve and 2 mm for the aortic valve. Mitral valve regurgitation was graded based on the extent of the regurgitant jet into the left atrium (LA) as follows: Grade 1 (jet extending up to proximal ¼ of the LA), grade 2 (½ way up the LA), grade 3 (up to ¾ of the LA), and grade 4 (beyond ¾ of LA).

Pulmonary hypertension definition by transthoracic echocardiography (TEE) was categorized as follows: normal pulmonary artery systolic pressure (PASP ≤ 35 mmHg), mild (PASP 36-45 mmHg), moderate (PASP 46-60 mmHg), and severe PH (PASP > 60 mmHg) [17].

Data were analyzed using IBM SPSS Statistics for Windows, version 26 (released 2019; IBM Corp., Armonk, New York, United States). Baseline sociodemographic characteristics for categorical variables were presented as count and percentage, while continuous variables were presented as mean and standard deviation. The prevalence of left ventricular systolic dysfunction (LVSD), left ventricular diastolic dysfunction (LVDD), left ventricular hypertrophy (LVH), pericardial effusion, pulmonary hypertension, and valvular abnormalities was determined. Logistic regression was used to determine the association between SLE disease activity index (SLEDAI), SLE duration, and prevalence of various cardiac abnormalities. The odds ratio (OR) and their 95% confidence intervals (CIs) were determined. P-values less than 0.05 were judged as statistically significant.

## Results

Baseline characteristics

Ninety-nine (99) SLE patients with no history of cardiac disease were enrolled in this study. The study group included 90 (90.9%) females and 9 (9.1%) males. The mean (±SD) age of the study population was 35.12 (±12.62) years. The majority (76, 76.8%) were between the ages of 18 and 39 years, followed by 40-59 years. Thirty-seven (37.4%) were overweight, while 40 (40.4%) were normal (Table 1).

**Table 1:** Demographic characteristics BMI: body mass index, ANA: antinuclear antibodies

Variables	N (%)
Age	
18-39	76 (76.8)
40-59	19 (19.2)
60 and above	4 (4.0)
Sex	
Male	9 (9.1)
Female	90 (90.9)
BMI status	
Underweight (<18.5 kg/m^2^)	6 (6.1)
Normal (18.5-24.9 kg/m^2^)	40 (40.4)
Overweight (25-29.9 kg/m^2^)	37(37.4)
Obese (30 and above kg/m^2^)	16(16.2)
ANA values	
Negative	2 (2.0)
1:80	1 (1.0)
1:160	74 (74.7)
1:320	13 (13.1)
1:640	9 (9.1)

Age at diagnosis, duration of SLE, and SLEDAI-2K score

The mean (±SD) age at diagnosis of SLE was 28.7 (±11.9) years. The calculated mean SLEDAI-2K score was 9.1 (±7.8). The mean SLE disease duration was 4.6 (±3.9). The mean duration of SLE treatment was 4.1 (±3.6) years (Table 2).

**Table 2:** Mean distribution of SLE duration and SLEDAI-2K score SLE: systemic lupus erythematosus, SLEDAI-2K: SLE Disease Activity Index 2000, SD: standard deviation

	Mean (± SD)	Minimum	Maximum
Age at diagnosis (years)	28.7 (±11.9)	11	72
Duration of SLE	4.6 (±3.9)	0	18
Duration of SLE medications (years)	4.1 (±3.6)	2 months	17
SLEDAI-2K score	9.1 (±7.8)	0	25

Table 3 shows the mean scores for the SLEDAI-2K score, age at diagnosis, SLE, and medication duration for high and low disease activities. The mean SLEDAI-2K score was significantly higher in participants with high disease activity, compared to those with low disease activity (p < 0.001). The mean ages at SLE diagnosis (p = 0.609), duration of SLE (p = 0.709), and duration of SLE treatment (p = 0.918) in the participants with a high disease activity were however not significantly different from those with a low disease activity.

**Table 3:** SLEDIA-2K score, age at diagnosis, SLE, and medication duration vs. disease activity status SLE: systemic lupus erythematosus, SLEDIA-2K: SLE Disease Activity Index 2000, SD: standard deviation

Parameters	Disease Activity; mean ± SD		t-test	p-value
	High	Low		
SLEDAI-2K score	13.8±5.8	1.0±0.3	12.74	<0.001
Age at SLE diagnosis	29.1±13.16	27.9±9.5	0.51	0.609
Duration of SLE (in years)	4.7±3.8	4.4±0.69	0.38	0.709
Duration of SLE medications (in years)	4.1±3.3	4.1±4.0	-0.10	0.918

All participants were on medication. Forty (40.4%) patients were on the combination of prednisolone, hydroxychloroquine, and azathioprine, 33 (33.3%) on prednisolone with hydroxychloroquine, and others with medications comprising prednisolone, hydroxychloroquine, methotrexate, mycophenolate mofetil, tacrolimus, or prednisolone alone (Table 4).

**Table 4:** SLE medications SLE: systemic lupus erythematosus, PPI: proton pump inhibitor

	Number	Percentage (%)
SLE medication taken		
Pred/hydroxy/azathioprine	40	40.4
Prednisolone and hydroxychloroquine	33	33.3
Others (either of the above medications alone)	26	26.3
Supplementary medication		
Proton pump inhibitor (omeprazole, esomeprazole)	80	87.9
Hematinics (blood tonic/pills)	2	2.2
PPI/hematinics	9	9.9

Prevalence of echocardiographic abnormalities

As shown in Table 5, 56 (47%) patients were found to have echocardiographic abnormalities. Valvular abnormalities were detected in 10 (8.7%) patients with mild mitral regurgitation being the most common finding. One patient (1%) had pericardial involvement, namely, pericardial thickening with mild pericardial effusion. Eighteen (15.7%) had LVDF, one (0.9%) patient had LVSD, and six (5.2%) patients had LVH. Of the patients who had diastolic dysfunction, 10 (8.7%) had grade I diastolic dysfunction, seven (6.1%) had grade II diastolic dysfunction, and one (0.9%) had grade III diastolic dysfunction. Fourteen (12.2%) patients had pulmonary hypertension, 13 (11.3%) had mild pulmonary hypertension, and one (0.9%) had moderate pulmonary hypertension. The mean (±SD) pulmonary hypertension was 28.9 (±5.5).

**Table 5:** Echo abnormalities

Subclinical echo abnormalities	Frequency	Percentage (%)
Pericardial involvement	1	1.0
Mild pericardial effusion and pericardial thickening	1	1.0
Valvular	10	8.7
Mild aortic regurgitation	1	0.9
Mild pulmonary regurgitation	1	0.9
Mild mitral regurgitation	5	4.3
Mild tricuspid regurgitation	2	1.7
Moderate mitral regurgitation	1	0.9
Left ventricular diastolic dysfunction	18	15.7
Grade I	10	8.7
Grade II	7	6.1
Grade III	1	0.9
Left ventricular systolic dysfunction	1	0.9
Pulmonary hypertension	14	12.2
Mild	13	11.3
Moderate	1	0.9
Left ventricular hypertrophy	6	5.2
Total	56	47

The LV mass indexed to the body surface area showed that six (6.7%) of the female participants had elevated left ventricular mass index above the reference value (>95 g/m^2^), as shown in Table 6.

**Table 6:** Left ventricular mass index Left ventricular mass index (LVMI): referenced to the body surface area (BSA) g/m^2^

	Male: n (%)	Female: n (%)
Normal	9 (100)	84 (93.3)
Mildly abnormal	0 (0.0)	2 (2.2)
Moderately abnormal	0 (0.0)	1 (1.1)
Severely abnormal	0 (0.0)	3 (3.3)
Total	9 (100)	90 (100)

Echocardiographic abnormalities and profile of SLE disease activity

**Figure 1:**
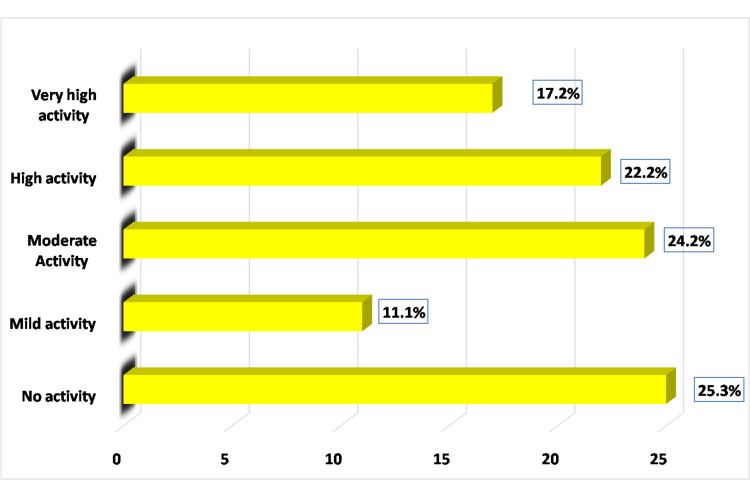
Systemic lupus erythematosus (SLE) disease activity classification

Disease activity was classified as no activity, mild, moderate, high, and very high disease activity based on the SLEDAI-2K score. Details of the SLEDAI-2K scoring system are presented in Figure 1. Thirty-five patients (35.3%) had mild to moderately active disease, with mean age of 32.1 ± 11.9 years and mean disease duration of 5.1 ± 4.3 years. Thirty-nine (39.4%) had severely active disease (high and very high activities), with their mean age of 36.7 ± 13.5 years and mean disease duration of 5.1 ± 4.1 years (Figure 1).

It was observed that there was a significant association between disease activity and echocardiographic abnormalities. Out of the patients who had low disease activity, three (8.6%) had abnormal echocardiographic findings, while 36 (56.2%) out of the severely active group had abnormal echocardiographic findings (Table 7).

**Table 7:** Association between disease activity and echocardiographic abnormalities in the systemic lupus erythematosus (SLE) patients

	Disease activity			
	Low	High	Chi-square value	P-value
Normal echo (%)	32 (91.4)	28 (43.8)	21.54	<0.001
Abnormal echo (%)	3 (8.6)	36 (56.2)		

From the logistic regression analysis, the odds of echocardiographic abnormalities was 13.7 times higher in SLE patients with high disease activity compared to those with low disease activity (OR = 13.714, 95% CI = 3.804-49.442, p < 0.001). SLE patients with high disease activity were more likely to have echocardiographic abnormalities compared to their counterparts with low disease activity, even after adjusting for potential confounders (adjusted OR (aOR) = 10.206, 95% CI = 2.714-38.381, p = 0.001) (Table 8).

**Table 8:** Logistic regression analysis of disease activity and echocardiographic abnormalities in SLE patients Model 1 was unadjusted; Model 2 was adjusted for age and systolic and diastolic blood pressures. SLE: systemic lupus erythematosus, OR: odds ratio, CI: confidence interval

Variable	OR	95% CI	p-value
Model 1	13.714	3.804-49.442	<0.001
Model 2	10.206	2.714-38.381	0.001

Association between disease activity and specific echocardiographic abnormalities

Table 9 shows the association between specific echocardiographic abnormalities and disease activity. It was observed that there were no significant associations of disease activity with the specific echocardiographic abnormalities. Seven (19.4%) of those with severe disease activity had valvular abnormalities. There was no significant association between those who had pulmonary hypertension and severe disease activity, even though 36.1% with severe disease activity had pulmonary hypertension (p-value = 0.923). About 3% of the participants with severe disease activity had pericardial involvement; however, the association was not statistically significant (p-value = 0.202).

**Table 9:** Association between echocardiographic abnormalities and disease activity SLEDAI: Systemic Lupus Erythematosus Disease Activity Index, LVH: left ventricular hypertrophy, LVDD: left ventricular diastolic dysfunction

Variable	Disease activity		Chi-square	p-value
	Low SLEDAI (no activity and mild activity)	High SLEDAI (moderate activity, high and very high activity)		
Valvular			0.711	0.3991
Present	0 (0.0)	7 (19.4)		
Absent	3 (100)	29 (80.6)		
Pulmonary hypertension			0.009	0.923
Present	1 (33.3)	13 (36.1)		
Absent	2 (66.7)	23 (63.9)		
Pericardial involvement			1.625	0.202
Present	0 (0.0)	1 (2.8)		
Absent	3 (100)	35 (97.2)		
LVH			2.374	0.123
Present	2 (66.7)	9 (25.0)		
Absent	1 (33.3)	27 (75.0)		
LVDD			2.031	0.154
Present	0 (0.0)	15 (41.7)		
Absent	3 (100)	21 (58.3)		

Echocardiographic abnormalities and disease duration

There were 57 (57.6%) SLE patients whose disease duration was less than five years, while 42 (42.4%) were five years and above with mean age of 32.5 ± 11.2 and 34.2 ± 12.5 years, respectively. Of those with SLE whose disease duration was less than five years, 23 (40.4%) had abnormal echocardiographic findings, while 16 (38.1%) patients with a disease duration of 5 years and more had abnormal echocardiographic findings. It was found that there was no significant association between echocardiographic findings and duration of disease (p = 0.766) (Table 10).

**Table 10:** Association between echo findings and disease duration

SLE disease duration	Echocardiographic findings; n (%)		Chi-square test	p-value
	Normal	Abnormal	0.089	0.766
<5 years	33 (58.9)	23 (41.1%)		
≥5 years	26 (61.9)	16 (38.1)		

There was also no significant association between echocardiographic abnormality parameters and SLE duration (p > 0.05). It was also found that participants with SLE duration of five years and above were more likely to have LVH, LVDD, and valvular diseases compared to those with SLE duration less than five years (Table 11). 

**Table 11:** Association between SLE duration and echo abnormalities LVDD: left ventricular diastolic dysfunction, LVH: left ventricular hypertrophy, OR: odds ratio, CI: confidence Interval

Variable	SLE duration		Chi-square	p-value
	<5 years	≥5 years		
Valvular			3.467	0.063
Present	11 (47.8)	3 (18.8)		
Absent	12 (52.2)	13 (81.3)		
Pulmonary hypertension			0.714	0.398
Present	1 (4.3)	0 (0.0)		
Absent	22 (95.7)	16 (100)		
Pericardial Involvement			0.124	0.724
Present	6 (26.1)	5 (31.3)		
Absent	17 (73.9)	11 (68.8)		
LVH			3.627	0.057
Present	6 (26.1)	9 (56.3)		
Absent	17 (73.9)	7 (43.8)		
LVDD			0.012	0.913
Present	4 (17.4)	3 (18.8)		
Absent	19 (82.6)	13 (81.3)		

Table 12 shows that the participants on prednisolone/hydroxychloroquine/azathioprine were more likely to develop LVDD and valvular issues. None of these associations were statistically significant.

**Table 12:** Association between echocardiographic abnormalities and SLE medications LVDD: left ventricular diastolic dysfunction, LVH: left ventricular hypertrophy, OR: odds ratio, CI: confidence Interval, SLE: systemic lupus erythematosus

	Pred/Hydroxy/azathioprine OR (95% CI); p-value	Prednisolone and hydroxychloroquine OR (95% CI); p-value
Pulmonary hypertension	0.37 (0.9-1.47); 0.370	0.46 (0.12-1.84); 0.460
Pericardial	0 (0.0); 0.998	0 (0.0); 0.998
LVH	0.97 (0.15-6.26); 0.977	2.67 (0.49-14.48); 0.256
LVDD	1.35 (0.31-5.96); 0.690	1.70 (0.38-7.58)
Valvular issues	1.33 (0.23-7.86); 0.751	0.38 (0.03-4.38)

There was no significant association between SLE treatment duration and echocardiographic abnormalities in the SLE patients (OR = 1.074, 95% CI = 0.908-1.269, p = 0.405). It was also observed that there was no significant association between SLE treatment duration and the specific echocardiographic abnormalities in the SLE patients (p > 0.005) (Table 13).

**Table 13:** Association between echocardiographic abnormalities and SLE treatment duration LVDD: left ventricular diastolic dysfunction, LVH: left ventricular hypertrophy, OR: odds ratio, CI: confidence Interval, SLE: systemic lupus erythematosus

	OR (95% CI)	p-value
Pulmonary hypertension	0.82 (0.65-1.04)	0.097
Pericardial	0.44 (0.06-3.23)	0.417
LVH	0.96 (0.79-1.16)	0.647
LVDD	1.07 (0.93-1.24)	0.349
Valvular	1.04 (0.85-1.27)	0.696

Table 14 shows as the age increased, the participants were more likely to develop echocardiographic abnormalities except for pericardial. This was found to be significant in those who developed LVDD.

**Table 14:** Association between echocardiographic abnormalities and age LVDD: left ventricular diastolic dysfunction, LVH: left ventricular hypertrophy, OR: odds ratio, CI: confidence Interval

	OR (95% CI)	p-value
Pulmonary hypertension	1.02 (0.97-1.07)	0.426
Pericardial	0.0 (0.0)	0.978
LVH	1.038 (0.99-1.09)	0.116
LVDD	1.05 (1.01-1.10)	0.017
Valvular	1.01 (0.95-1.07)	0.783

## Discussion

This study looked at cardiac abnormalities in SLE patients detected using 2D echocardiography and their relationship with SLE disease activity measured using the modified SLEDAI-2K. The SLEDAI-2K was chosen as the tool for measuring disease activity because it is extremely practical, reliable, accurate, and cheap and therefore widely used for research purposes. The study aims to determine the prevalence of these echocardiographic abnormalities and ascertain their association with disease activity. The prevalence of electrocardiographic abnormalities in these patients was also determined, and their association with disease activity was ascertained.

Cardiovascular diseases have been well documented as a recognized cause of morbidity and mortality in SLE. Cardiac involvement in recent times is frequently, documented due to the longer survival of SLE patients as a result of better control of disease activity and infection in recent years [18].

In this study population, 47% of the patients had echocardiographic abnormalities, with 8.7% showing valvular abnormalities (comprising mild incompetence of the aortic, pulmonary, mitral, and tricuspid valves). One patient, however, had moderate mitral regurgitation, 15.7 had diastolic dysfunction, and 12.2% had pulmonary hypertension (most of the patients had mild pulmonary hypertension with one patient having moderate pulmonary hypertension). LVH was seen in 5.2%, 0.9% had LV systolic dysfunction, and 1% with pericardial involvement. No significant correlation was found between echocardiographic cardiac abnormalities and disease activity as well as disease duration.

The overall prevalence of cardiac abnormalities in this study is similar to that in Cervera et al.’s prospective echo study of 70 SLE patients, which revealed that 57% of the patients exhibited echocardiographic abnormalities [19]. The study used similar echocardiographic modalities and documented a similar range of cardiac abnormalities. In the study by Zhang et al., echocardiographic abnormalities were found in 53.3% of patients, with 38.9% having valvular abnormalities and 34.4% having pericardial effusion [20].

In a meta-analysis done among SLE patients in native sub-Saharan Africa, cardiovascular involvement was found in 2-46%, comprising 7.8-30.8% having pericardial abnormalities, 4.3-33% heart failure, 5.7-20% LVH, 16% with pulmonary hypertension, and 7.7-8.7% with myocarditis [21].

The prevalence of valvular disease in this study (8.7%) is close to the range (13-50%) found in some autopsy studies. Abnormalities of the mitral valve have been documented to be the predominant valvular lesion seen in SLE patients followed by the tricuspid valve [22]. Bourre-Tessier et al. also found the mitral valve to be the most commonly affected valve with 25.4% of their patients having mitral valve thickening and 25.8% with mitral regurgitation [23]. In the meta-analysis by Hussain et al., the most commonly involved valve was the mitral valve; 19.7% had mitral regurgitation, 11.06% valve thickening, and 11.70% vegetation [24]. This was replicated in our study, where most of our study participants presenting with valvular abnormalities had mitral involvement, mainly mitral regurgitation, followed by tricuspid regurgitation. In one study done among 50 asymptomatic SLE patients, mitral regurgitation was found in 32% of patients, pericardial effusion in 32%, aortic regurgitation in 10%, and tricuspid regurgitation in 2% [2].

Moder et al. suggested that valvular regurgitation may be due to a combination of factors, such as the presence of Libman-Sacks endocarditis, previous rheumatic fever, fibrinoid degeneration, fibrosis, valvulitis, and bacterial endocarditis [25].

In the study by Conteh et al., in Kenya, the mitral valve was the most commonly affected valve. A portion (69.8%) had mitral valve thickening and 30.2% had mitral regurgitation [26]. The overall prevalence of echocardiographic abnormalities in their study population was 88.9%, comprising pericardial, myocardial, valvular, and pericardial abnormalities [26]. The investigators, however, stated that the high prevalence found may have been due to clinically insignificant pericardial thickening and valve thickening [26]. However, this study did not find any patients with valvular thickening. Some of these differences in the cardiac findings may be partly due to different patient characteristics, such as age, racial differences, disease phenotype, and antibody profiles [27,28].

Various authors have sought to investigate the correlation between the valvulopathy seen in SLE and disease duration. Most of them did not find any strong evidence for the correlation between disease duration, patient age, and disease severity with valvular involvement in SLE. Mohammed et al. in their study, however, found that SLEDAI was associated with an increased risk of mitral valvular leaflet thickening [29]. 

The prevalence of pulmonary hypertension found in this study was 12.2%. The prevalence of pulmonary arterial hypertension (PAH) in SLE has not been well established, but some epidemiological studies have established a prevalence between 0.5% and 17.5%. The wide differences in prevalence have been explained by the different patient selection criterion used in the various studies and the different diagnostic techniques used, such as Doppler echocardiography or right heart catheterization. Conteh et al. in their study, for example, had a higher prevalence of pulmonary hypertension, which was 22.2% [26]. The prevalence of pulmonary hypertension in these studies is important because of the significant morbidity and mortality associated with pulmonary hypertension in patients with SLE.

Most of our study population had mild pulmonary hypertension defined as PASP between 20 and 40 mmHg. The mean (±SD) PASP was 28.9 (±5.5). This is similar to the findings of the meta-analysis done by Xia et al., where they noted that most of the cases of SLE-associated pulmonary hypertension are mild, making early diagnosis difficult [30]. In the prospective study by Simonas et al., done to determine the prevalence and severity of pulmonary hypertension in SLE patients, they found a prevalence of 14%, which is similar to this study with their mean PASP being 25 ± 10 mmHg. This confirms the suggestion that pulmonary hypertension in SLE is common but usually mild. Over 40% of SLE patients with mild pulmonary hypertension tend to have no symptoms [31]. Moreover, the clinical symptoms of pulmonary hypertension, such as dyspnea and fatigue, tend to be non-specific and can easily be missed, hence the importance of early detection. TTE is a widely used tool used to screen for pulmonary hypertension because of its safety, convenience, and highly documented sensitivity.

Results from various studies have proven that SLE patients with pulmonary hypertension tend to have a poorer prognosis than those without pulmonary hypertension. Various mechanisms have been proposed to contribute to the pathophysiology of PH in SLE with immune and inflammatory mechanisms playing a significant role, both in the pathogenesis and progression of PAH in SLE. Fibroblast and endothelial cell dysfunction, resulting in the impaired production of vasodilators, such as nitric oxide and overproduction of the vasoconstrictors; mainly, endothelin leads to the remodeling of the pulmonary vasculature and, thus, PAH. Studies have also documented the presence of elevated serum levels of pro-inflammatory cytokines and growth factors in the pulmonary arteries of patients with PAH [30,31]. The presence of vasculitis and interstitial pulmonary fibrosis may also lead to endothelial and smooth muscle proliferation and damage of the pulmonary vessels, leading to pulmonary hypertension. In a study by Lian et al., they found that the leading predictors of SLE-associated PAH are Raynaud’s phenomenon, positive anti-cardiolipin, or anti-U1 ribonucleoprotein (RNP) antibody and the presence of serositis [32]. Assessment of the pulmonary artery pressure and right heart function, using echocardiography, is therefore recommended, especially in SLE patients with these associated factors.

The pericardial disease has for a long time been known to be one of the commonest cardiac manifestations of SLE, presenting either as effusions (indicative of active pericarditis) or pericardial thickening as sequelae of active disease. It is also the most common echocardiographic lesion in SLE patients and the most frequent cause of symptomatic disease. In this study, only one of the participants had pericardial involvement (1.0%), which comprised mild pericardial effusion with pericardial thickening. Recent transthoracic echocardiographic studies have found pericardial involvement in 0-27% of patients, occurring mostly as effusions [33]. In a prospective study of 70 SLE patients, done by Cervera et al., 27% had pericardial abnormalities, while in another study, 25.9% of the study population had pericardial involvement [19]. Shahin et al in Egypt had 19% of their patients having pericardial effusion [34]. The low prevalence of pericardial involvement and pericardial effusion seen in this study may be because all of our patients were on corticosteroids or two or more disease-modifying drugs at the time of the study. This may have minimized the active inflammation in the pericardial tissue. Another reason for our low prevalence could be that our inclusion criteria excluded symptomatic patients.

The prevalence of LVH in this study was 5.2% (six patients). This finding is similar to that seen in studies by Cerveva et al. [19] and Omdal et al. [33], where they reported six out of 70 and three out of 35 SLE patients, respectively. Ventricular remodelling, leading to LVH, may be due to inflammation-related vascular stiffness. Subclinical coronary artery disease has also been found to contribute to structural changes of the left ventricle, such as LVH, as a result of the premature atherosclerosis seen in SLE. Even though hypertension is a well-known stimulus of LVH, SLE patients have been reported to develop LVH independent of hypertension, suggesting a direct disease-related effect of SLE on the LV structure [35]. Findings of LVH among SLE patients documented in some studies were confounded by coexisting hypertension and valvular or renal disease. In this study, patients with hypertension and other cardiovascular disease were excluded to limit the effect of these risk factors on the findings.

In our study, almost all the patients had good left ventricular systolic function except one patient (0.9%) who had severe LVSD. Our low prevalence of systolic dysfunction is similar to the study by Shazzad et al. in Bangladesh who reported systolic dysfunction in 8% of SLE patients [22] and De Godoy et al. who had 3.8% of their study group with systolic dysfunction [36]. Conteh et al. in Kenya also found that 11.1% of their patients had mild systolic dysfunction [26].

Yip et al. noted in their study that SLE patients with reduced LV systolic function had a longer duration of the disease (over 10 years) and increased disease activity [37]. It is therefore very important to evaluate LV systolic function in SLE patients with long disease duration or increased disease activity. Chen et al. also found that prolonged disease duration (notably more than 10 years) and high disease activity score (SLEDAI/Systemic Lupus International Collaborating Clinics (SLICC)) are associated with both systolic and diastolic dysfunctions [38]. Systolic dysfunction in SLE patients may be a result of myocarditis, severe valvular disease, or subclinical coronary artery disease due to accelerated atherosclerosis found in SLE patients [39]. Myocardial involvement in SLE has been reported in some pathological studies to be between 40% and 70%; however, it is well known that the ventricular dysfunction that occurs as a result of myocardial involvement tends to be clinically silent.

In this study, the prevalence of LVDD is 15.7%. This is similar to that reported by Nikdoust et al., which was 16% [40], and Ong et al., who found that 10% of their SLE patients had LVDD [41]. Studies have shown that LVDD largely tends to be the only finding of cardiac involvement preceding global systolic dysfunction. Diastolic dysfunction in SLE patients affects their quality of life, exercise tolerance, and overall prognosis; thus, early detection is vital. 

Some studies have reported an association between disease severity and diastolic dysfunction in SLE patients. Wilowska et al., in their study done to assess LV systolic and diastolic functions in asymptomatic SLE patients, also sorted to establish whether there is a correlation between the duration and severity of SLE and the degree of LVDD [42]. They found a significant increase in LVDD among SLE patients compared to controls and especially those with disease duration more than 10 years [42]. The patients with LVDD had higher SLEDAI scores, but there was no statistically significant correlation between SLEDAI and LVDD. Shang et al. also found that patients with SLICC/American College of Rheumatology (ACR) damage index scores of ≥1 had more impaired LVDD compared to those without it [37].

Nikdoust et al. also noted an increase in left atrial and LV end-diastolic volume, indicating diastolic dysfunction in patients with severely active SLE than those with mild to moderately active SLE [40]. Aortic stiffness is independently correlated with LVDD in young adult SLE patients, according to Roldan et al. [43]. Aortic stiffness causes an increase in the LV afterload and LV mass, leading to decreased coronary perfusion and, subsequently, LVDD.

Roldan et al.'s study also noted that certain categories of SLE patients have an increased risk of developing LVDD, and this includes those with longer SLE duration, severe SLE (as determined either by SLEDAI or damage score), and higher levels of acute-phase reactants, such as C3 levels [43]. This, therefore, suggests that early diagnosis of SLE with prompt initiation of anti-inflammatory and immunosuppressant therapy may slow down the progression of diastolic dysfunction and diastolic heart failure.

There was a significant association between increasing age and LVDD in this study. This is not surprising as age is well known to be associated with diastolic dysfunction due to increasing arterial stiffness associated with aging. Studies have shown that this age-related arterial stiffness is more common in women than in men; thus, with the larger number of the study participants being women, this association is not surprising.

Mild to moderately active disease was seen in 35.3% of the participants, while 39.4% had severely active disease. A portion (66.7%) of the severely active group had abnormal echocardiographic findings, while 26.6% of those with mild to moderately active disease had echocardiographic abnormalities. There was no significant correlation between SLE disease activity and echocardiographic abnormalities. This notwithstanding, participants with high SLEDAI-2K scores were more likely to develop valvular abnormalities, pulmonary hypertension, and left ventricular hypertrophy than those with low SLEDAI. This is similar to the studies by Mohammed et al. [8] and Ong et al. [41] who found no significant association between disease activity and the prevalence of echocardiographic abnormalities. Generally, the results regarding the association between disease activity and cardiac abnormalities have been conflicting. In one study, PAH and myocarditis had a significant correlation with disease activity but not valvular or pericardial involvement. Another study found a statistically significant correlation between SLEDAI scores and LVDD. A statistically significant correlation between SLEDAI and pericardial effusion has also been demonstrated by several studies [2,10,44]. Yu et al. also reported that disease duration and disease activity have a significant association with the presence of cardiac abnormalities [45]. Paran et al. also found no association between disease activity with impaired systolic or diastolic function, but those with high SLEDAI scores had increased left atrial and ventricular size [39].

There was also no significant association between echocardiographic abnormalities and SLE disease duration in this study. However, those with a disease duration of more than five years were more likely to have LVH, LVDD, and valvular abnormalities. These findings are similar to the study done by Ong et al., where they could not demonstrate any correlation between the prevalence of cardiac disease and SLE disease activity or duration [41]. Lolli et al. also did not find any significant association between disease duration and echocardiographic abnormalities [46]. This study’s findings are also similar to other studies that showed a positive correlation between disease duration and LVH, diastolic dysfunction, and valvular abnormalities [45]. One study showed that the independent predictors of LVSD include a long disease duration of more than 10 years and a high disease activity index, suggesting that disease chronicity with SLE flares leads to significant systolic impairment [37]. Another author noted that younger patients with SLE tend to have an increased incidence of CV events, such as acute myocardial infarction, after a long disease duration [47].

Limitations

The study is limited by the following: First, the echocardiography examination was conducted by a single observer, and there may be risks of bias in interpreting the results. Second, although the SLEDAI is considered the gold standard for assessing disease activity in SLE patients, the modified SLEDAI-2K was used in this study because it eliminates the immunologic variables, which are very expensive and thus may not be readily accessible or available in our resource-poor setting. Lastly, although echocardiography is generally cheaper than some modern imaging modalities, such as cardiac CT scan and MRI, it is still relatively expensive in our population and thus may not be readily accessible to some patients in our setting.

## Conclusions

In asymptomatic SLE patients, echocardiography is a practical and reliable method for identifying subclinical heart dysfunction or abnormalities. In light of this, clinical outcomes in SLE patients may be predicted by changes in echocardiographic parameters. Cardiac abnormalities, especially LVDD and valvular involvement, are common in SLE patients with most being asymptomatic. Earlier diagnosis of SLE with prompt initiation of anti-inflammatory and immunosuppressive therapy slows down the progression of cardiac affectation, such as LVDD and, subsequently, diastolic heart failure.

We, therefore, recommend that SLE patients with long disease duration and high disease activity should be monitored to help identify those who may have cardiac abnormalities or those at risk of serious cardiac complications. More so, the screening of all cases of SLE with an echocardiogram should be done especially at presentation as a baseline echo and during flares to identify those with cardiac abnormalities for earlier management and reduction in morbidity and mortality.
